# Predicting professional school performance with a unique lens: are there other cognitive predictors?

**DOI:** 10.1186/s12909-020-1930-2

**Published:** 2020-01-15

**Authors:** Theresa A. Davies, Madeline B. Miller, Vincent A. Moore, Elizabeth A. Kaye

**Affiliations:** 1 Department of Medical Sciences & Education, Boston University School of Medicine, Boston, MA USA; 2 Graduate Medical Sciences, Boston University School of Medicine, 72 East Concord Street, L317, Boston, MA 02118 USA; 3 Department of Health Policy and Health Services Research, Henry M. Goldman Boston University School of Dental Medicine, Boston, MA USA

**Keywords:** Admissions criteria, Academic performance, Underrepresented groups, Pipeline, Dental, Medical, DAT

## Abstract

**Background:**

We investigated the associations between admissions criteria and performance in four cohorts of pre-dental MS in Oral Health Sciences (OHS) program at Boston University Schools of Medicine and Dental Medicine. Previously we have reported that OHS serves as a successful pre-dental pipeline program for students from underrepresented groups.

**Methods:**

We evaluated academic variables that further affect overall graduate GPA and grades in the first year dental school courses taken by OHS students at Boston University between 2012 and 2016 as part of the MS curriculum. Demographic data, region of residency, undergraduate grade point average, number of science and math credits, major of study, dental admissions test scores and undergraduate institution were collected. The competitiveness of the undergraduate institution was scored based on Barron’s Profiles of American Colleges. OHS-GPA was assessed and individual grades in two first year dental school courses taken as part of the OHS curriculum were collected. Analysis of variance, the Chi-square test and Fisher’s Exact test were utilized to assess associations between academic performance parameters, successful program completion and matriculation to dental school.

**Results:**

Results indicate that undergraduate major, age and number of science course credits taken had no impact on MS performance in the Boston University MS in Oral Health Sciences program; however, students who took an undergraduate course in Physiology performed better than those who did not (*p* = 0.034). This was not the case with courses in Cell Biology and Biochemistry. Students with DAT scores over 20 academic average (*p* = 0.001), 18 total science average (p = 0.001) and 22 reading comprehension (*p* = 0.004) performed better in dental school courses taken in OHS.

**Conclusion:**

We report that strong test scores, attending a mid or highly rigorous undergraduate institution and completion of an undergraduate Physiology course are positive predictors. We hope these findings will guide admission’s decisions and improve recruitment to, and future success of, graduate student’s pursuit of professional school. Understanding alternative predictors of success may help to reduce the intrinsic bias among applicants from underrepresented groups and continue to look beyond the DATs (or MCATs) to decrease the gap between professionals from underrepresented groups and those they serve.

## Background

Admission to medical and dental schools as well as doctoral programs in the biomedical sciences is extremely competitive. In hope of ensuring a successful candidate, professional schools have moved towards a holistic review and the use of a combination of cognitive and noncognitive attributes to assess applicants [1]. Cognitive domains encompass those that are quantitative (undergraduate GPA, standardized exams), while noncognitive domains are qualitative (interview, letters of recommendation, emotional intelligence). Unfortunately, many of these factors have been shown to have a statistically insignificant correlation with academic success and more longitudinal studies examining predictors of success after admission are needed.

Commonly, the first set of data evaluated by admissions committees are the cognitive skills. Standardized test scores such as the graduate record exam (GRE) and undergraduate GPA (UGPA) often are the determining factor for an applicant to progress within the admission system. Setting minimum criteria for admission based on test scores such as the GREs has shown modest consistency in reported studies over the past decade [2]. In dental admissions, studies have been mixed. A study from the University of Florida College of Dentistry of graduating classes from 1994 to 1999 [3] as well Harvard Dental School supported the positive correlation between the UGPA and success in dental school [4]. Similar results were reported by Lee et al. at Columbia University College of Dental Medicine [5] and Plouffe et al. at Schulich School of Medicine & Dentistry, University of Western Ontario, Canada [6]. Plouffe states that DAT chemistry scores serve as positive predictors of successful dental school performance. Rowland & Rieken evaluated six classes from 2007 to 2013 at Southern Illinois School of Dental Medicine and noted that while there was a strong correlation between overall UGPA and performance in dental school, they noted this only accounted for 40% of the variance in GPA among the first year of dental school. Other factors, such as science GPA and test section sub-scores, they reported, were important in assessing performance although they also noted that factors such as emotional intelligence were relevant as well [7]. They note that this correlation is not a significant determining factor because there were students that outperformed the prediction and students who significantly underperformed the projected dental school GPA based on this admission criteria [7]. This data is consistent with a study done at University of California, Davis School of Medicine that showed students who were admitted with a Medical College Admissions Test (MCAT) score and UGPA below the minimum requirement outperformed many of the individuals who were admitted who met the standard minimum requirements [8]. Park et al. evaluated performance in doctoral students in the biomedical sciences at Boston University and demonstrated a significant positive correlation between academic performance in year 1 of doctoral graduate study and undergraduate GPA and GRE scores [9]. They also used undergraduate institution rigor to normalize UGPA, a factor that is often not included when discussing UGPA.

Noncognitive abilities have been evaluated in a doctoral program at University of Ottawa Medical School however a clear correlation among these abilities and academic success were inconclusive [10]. These results are similar to those found by Plouffe et al. evaluating interview scores to assess communication, interpersonal skills, resiliency, and integrity [6]. They found there was no positive correlation between interview score and any other preadmission variable or dental school grades. On the contrary, emotional intelligence has been reported to positively correlate with clinical performance among students who communicate effectively and show interpersonal sensitivity in their interactions with patients [11]. Further, studies evaluating DAT test sub-scores with clinical performance have been less successful [8] .

While cognitive and noncognitive abilities are used as predictors for performance in most doctoral professional programs who strive for holistic review, there continues to be a need for further longitudinal studies on the contribution of both cognitive and noncognitive attributes in admissions selections. In the changing and evermore competitive academic environment, applicants have sought out academic enrichment master’s programs as a means to enhance their credentials for medical and dental school admission as well as enhance their professional development. The MS in Oral Health Sciences (OHS) program is one such program geared to strengthen an applicant’s credentials for dental school admission. The OHS program was developed in 2005 by Boston University Henry M. Goldman School of Dental Medicine in collaboration with Graduate Medical Sciences at Boston University School of Medicine. The 32-credit program over its first 10 years has graduated more than 200 students with more than 90% of its graduates matriculating to (enrolling in) dental school [12]. Additionally, more than 25% of OHS graduates have come from groups under-represented in the field of dentistry thus serving as a pipeline program [12]. Previous work demonstrated that a strong performance in the OHS master’s program (*p* < 0.001) is predictive of future dental school success as measured by matriculation to (or enrollment in) dental school and success in the first two years of didactics. These results are strong; however, we are still working to improve our admissions criteria in order to better predict student success both in OHS as well as subsequently in dental school. Here we continue this work by evaluating academic variables from both their undergraduate and graduate years that may serve as additional positive predictors for future dental school success including undergraduate school rigor and specific coursework.

## Methods

### Ethics

The study was an analysis of de-identified data. This study was determined to be EXEMPT by the Institutional Review Board of Boston University Medical Campus, Protocol # H-33295.

### Data collection

Data for students enrolled in the OHS program at Boston University between 2012 and 2016 were utilized. Data on their gender, age, region of residency, undergraduate grade point average (GPA), number of science (Biology, Chemistry, Physics (BCP)) and math credits (BCPM), major of study, dental admissions test (DAT) scores and undergraduate institution attended were collected from admissions records to the master’s program. The competitiveness of the undergraduate institution was scored based on Barron’s Profiles of American Colleges on a scale of one to three, with one representing the most competitive institution [13].

Academic performance in the OHS graduate program was collected from the Graduate Medical Sciences Registrar. Overall OHS-GPA was assessed as well as individual grades in two first year dental school courses (OH 730 Physiology, OH 751 Biochemistry) taken as part of the OHS curriculum. These two courses are BU School of Dental Medicine year one courses and OHS students, as part of their graduate curriculum, enroll in these courses to demonstrate academic readiness for the rigors of dental school. OHS student sit side-by-side first year BU sdental students. If the OHS students took the DAT more than once, the best score was used in the analysis.

### Data analysis

Analysis of variance, the Chi-square test and Fisher’s Exact test were utilized to assess associations between academic performance parameters, successful program completion and enrollment in dental school.

Multivariable linear and logistic regression models were constructed to identify the most important independent predictors of OHS-GPA and performance in first year dental school courses, respectively. The models initially included variables that were significantly associated with those outcomes in bivariate analyses. These variables were age, gender, undergraduate school rigor, undergraduate school region, undergraduate physiology course, DAT total science score, and DAT reading comprehension score. Selection of the final models was performed in a stepwise manner and variables were allowed to stay in the model if their *P* value was < 0.05.

## Results

### Demographics and undergraduate credentials

Pre-admissions data was collected for 129 enrolled OHS master’s students (Table 1); gender breakdown was slightly more female, most enrollees had attended an undergraduate institution grouped in the mid or highly rigorous category and the majority chose to major in a science. Approximately 50 % of enrollees to the OHS program had previously applied to dental school and were unsuccessful in gaining admission. The cohort was 8% African American, 27% Asian, 20% Latino/Hispanic, 1% Native American and 44% White. Average undergraduate GPA was 3.10 ± 0.26 while DAT scores were competitive ranging from 18.8 to 21 for academic average, reading comprehension and total science sub scores.

**Table 1:** Characteristics of OHS cohort upon entry into program (*N* = 129). Mean ± SD or %

Age	24 ± 2
Gender	
Female	72 (56%)
Male	57 (44%)
Race / Ethnicity	
African American	10 (8%)
Asian	35 (27%)
Latino/Hispanic	26 (20%)
Native American	1 (< 1%)
White	57 (44%)
Undergraduate School Rigor	
Low	23 (17%)
Mid	51 (40%)
High	55 (43%)
Undergraduate Major	
Biology, Chemistry, Physiology	92 (71%)
Health Sciences	12 (9%)
Anthropology, Human Behavior	6 (5%)
Engineering, Math	3 (2%)
Business	5 (4%)
Other	11 (9%)
Undergraduate School Region	
Massachusetts	35 (27%)
Other New England	15 (12%)
Mid-Atlantic, Maryland, Washington DC	23 (18%)
South	24 (19%)
Midwest	14 (11%)
West	17 (13%)
Outside US	1 (1%)
Undergraduate GPA	3.10 ± 0.26
# Undergraduate BCP credits	68 ± 20
# Undergraduate BCPM credits	80 ± 22
Took undergraduate Anatomy & Physiology course	57 (44%)
Took undergraduate Biochemistry course	105 (81%)
Took undergraduate Cell Biology course	77 (60%)
Took undergraduate Microbiology course	71 (55%)
Took undergraduate Physiology course	78 (61%)
Previously applied to dental school	69 (53%)
DAT Academic Average	19.6 ± 2.0
DAT Reading Comprehension	21.0 ± 2.4
DAT Total Science	18.8 ± 1.7
Previously applied to dental school	69 (54%)

### OHS student performance in dental classes by undergraduate predictors

The majority of students (58%) students earned a B+ or better in dental school Physiology with 7% failing while more than 65% performed better in dental school Biochemistry with comparable non-passing grades (6%) (Table 2).

**Table 2:** OHS Student performance in dental school courses taken as part of the OHS curriculum (N = 129) *Mean ± SD

OHS Physiology Grade (dental school course)	
A	16 (12%)
A-	15 (12%)
B+	44 (34%)
B	22 (17%)
B-	25 (19%)
C+	3 (2%)
C	3 (2%)
C-	1 (1%)
B+ or higher	75 (58%)
OHS Biochemistry Grade (dental school course)	
A	24 (19%)
A-	27 (21%)
B+	33 (26%)
B	24 (19%)
B-	15 (12%)
C+	4 (3%)
C	2 (2%)
B+ or higher	84 (65%)

Students from the mid-West compared to those from Massachusetts (*p* = 0.036) and younger students (< 23 year; *p* = 0.014) performed stronger academically (Table 3). When performance in dental school Physiology and Biochemistry were compared, both the male gender (*p* = 0.035) and students less than 23 years old (*p* = 0.026) performed significantly better than other cohorts (Table 3).

**Table 3:** Performance in OHS by undergraduate predictors † Means with matching symbol are significantly different (*N* = 129). Mean ± SD or %

	N	Mean OHS GPA (Mean ± SD)	% B+ or better grade in OHS Physiology	% B+ or better grade in OHS Biochemistry
Gender				
Female	72	3.49 ± 0.27	50	61
Male	57	3.51 ± 0.27	68	70
*P value*		*0.69*	*0.035*	*0.28*
Age				
< =23	79	3.55 ± 0.26	66	70
> 23	50	3.43 ± 0.26	46	58
*P value*		*.014*	*.026*	*.18*
Undergraduate Major				
Biology, Chemistry, Physiology	92	3.51 ± 0.27	58	67
Health Sciences	12	3.49 ± 0.26	50	67
Anthropology, Human Behavior	6	3.35 ± 0.26	50	50
Engineering, Math	3	3.57 ± 0.20	67	67
Business	5	3.42 ± 0.18	60	20
Other	11	3.60 ± 0.26	73	73
*P value*		*.53*	*.91*	*.35*
Undergraduate School Region				
Massachusetts	35	3.40 ± 0.26 †	46	63
Other New England	15	3.49 ± 0.25	53	53
Mid-Atlantic, Maryland, Washington DC	23	3.52 ± 0.22	70	65
South	24	3.56 ± 0.25	54	63
Midwest	14	3.65 ± 0.28 †	78	86
West	17	3.47 ± 0.30	59	65
Outside US	1	3.88	100	100
*P value*		*.036*	*.33*	*.65*
# Undergraduate BCP credits				
< =53	26	3.48 ± 0.25	54	50
54–66	36	3.46 ± 0.29	53	58
67–75	33	3.52 ± 0.25	64	73
> =76	34	3.54 ± 0.28	62	77
*P value*		*.64*	*.75*	*.11*
# Undergraduate BCPM credits				
< =66	32	3.48 ± 0.27	56	50
67–75	26	3.48 ± 0.31	54	62
76–87	38	3.54 ± 0.25	63	76
> =88	33	3.49 ± 0.25	58	70
*P value*		*.74*	*.89*	*.12*

### Relationship between DAT and OHS student performance in dental classes

Students with academic average DAT scores of 20 and above (*p* < 0.001), total science 18 and above average (p < 0.001) and reading comprehension 22 and above (*p* = 0.004) performed better in both dental school courses and overall in the OHS master’s program as measured by OHS GPA (Table 4). Conversely, undergraduate GPA was not associated with performance in OHS. Students attending a less rigorous undergraduate institution did not perform as well as students attending a higher or mid-range rigor college in both dental school courses and overall in the OHS program (Table 4).

**Table 4:** Performance in OHS by DAT, undergraduate coursework and school rigor predictors. (N = 129). Mean ± SD or %. ‡ Significantly different from lowest quartile/group

	N	Mean OHS GPA	% B+ or better grade in OHS Physiology	% B+ or better grade in OHS Biochemistry
DAT Academic Average				
< =18	37	3.36 ± 0.25	30	46
19	30	3.44 ± 0.26	43	57
20–21	44	3.59 ± 0.22 ‡	77	80
> =22	18	3.67 ± 0.25 ‡	94	83
*P value*		*< 0.001*	*< 0.001*	*.004*
DAT Reading Comprehension				
< =19	33	3.40 ± 0.28	33	55
20–21	48	3.47 ± 0.25	58	60
22	21	3.62 ± 0.20 ‡	76	81
> =23	27	3.60 ± 0.28 ‡	74	74
*P value*		*.004*	*.003*	*.15*
DAT Total Science				
< =17	27	3.29 ± 0.26	30	41
18	32	3.53 ± 0.24 ‡	59	66
19–20	48	3.53 ± 0.23 ‡	58	69
> =21	22	3.65 ± 0.25 ‡	91	86
*P value*		*< 0.001*	*< 0.001*	*.008*
Undergraduate GPA				
< =2.93	31	3.43 ± 0.23	48	65
2.94–3.10	32	3.53 ± 0.26	63	69
3.11–3.25	33	3.53 ± 0.24	61	61
> =3.26	33	3.52 ± 0.32	61	67
*P value*		*.35*	*.65*	*.91*
Undergraduate School Rigor				
Low	23	3.33 ± 0.29	22	44
Mid	51	3.49 ± 0.23	57	67
High	55	3.58 ± 0.25 ‡	75	73
*P value*		*< 0.001*	*< 0.001*	*.045*
Took undergraduate Anatomy & Physiology				
Yes	57	3.49 ± 0.28	63	68
No	72	3.51 ± 0.26	54	63
*P value*		*.66*	*.30*	*.48*
Took undergraduate Biochemistry course				
Yes	105	3.50 ± 0.26	58	66
No	24	3.50 ± 0.30	58	63
*P value*		*.96*	*.98*	*.77*
Took undergraduate Cell Biology course				
Yes	77	3.51 ± 0.28	62	69
No	52	3.49 ± 0.25	52	60
*P value*		*.64*	*.24*	*.28*
Took undergraduate Physiology course				
Yes	78	3.54 ± 0.26	63	74
No	51	3.44 ± 0.27	51	51
*P value*		*.034*	*.18*	*.007*

Students who enrolled in an undergraduate Physiology course performed better in dental school courses and overall in the OHS program. This was not the case with undergraduate courses in Anatomy & Physiology, Cell Biology or Biochemistry (*p* = 0.034) (Table 4).

### Relationship between undergraduate and OHS student performance and matriculation to dental school

A large number of OHS graduates matriculated to BU School of Dental Medicine (67%) with Tufts accepting 12, and 17% attending other schools across the country including Meharry, New York University, University of New England, Detroit Mercy and Columbia. There were eight students or 6% who did not gain admission to any dental school. The dental school matriculation rate for the cohort was 94%.

Age (*p* = 0.32) and gender (*p* = 0.73) did not correlate with admissions to dental school however fewer students matriculated to dental school from less rigorous schools (*p* = 0.034) (Table 5). There was however a correlation between students earning a B+ or better in both dental school courses (Biochemistry *p* = 0.003 and Physiology *p* = 0.009) as well as overall GPA in the OHS program (*p* < 0.001) (Table 5).

**Table 5:** Dental School Admission by Predictors (N = 129) Mean ± SD or %

	Matriculated into dental school		
	Yes	No	P value
N	121	8	
Undergraduate School Rigor			
Low	83%	17%	.034
Mid	94%	6%	
High	98%	2%	
OHS GPA	3.53 ± 0.25	3.06 ± 0.17	< 0.001
Undergraduate GPA	3.09 ± 0.26	3.21 ± 0.28	.23
# Undergraduate BCP credits	67.7 ± 19.9	66.3 ± 23.3	.73
# Undergraduate BCPM credits	80.0 ± 21.3	76.8 ± 28.7	.63
Took undergraduate Anatomy & Physiology course			
Yes	93	7	.73
No	94	6	
Took undergraduate Biochemistry course			
Yes	94	6	.64
No	92	8	
Took undergraduate Cell Biology course			
Yes	94	6	.99
No	94	6	
Took undergraduate Physiology course			
Yes	94	6	.99
No	94	6	
% B+ or better grade in OHS Physiology			
Yes	99	1	.009
No	87	13	
% B+ or better grade in OHS Biochemistry			
Yes	99	1	.003
No	84	16	
DAT Academic Average	19.6 ± 2.0	18.5 ± 1.4	.11
DAT Reading Comprehension	21.0 ± 2.5	19.8 ± 1.2	.02
DAT Total Science	18.9 ± 1.7	17.9 ± 1.5	.12

### Multivariable regression

The significant independent predictors of OHS-GPA were undergraduate school rigor (*P* < 0.001), DAT total science score (P < 0.001), and DAT reading comprehension score (*P* < 0.05). Having attended a mid-rigor school compared to a low-rigor school resulted in an average increase in OHS-GPA of 0.12 ± 0.06 whereas attending a high rigor school increased OHS-GPA by 0.20 ± 0.06. Each 5-point increment of the DAT total science score increased OHS-GPA by 0.29 ± 0.07 and each 5-point increment of the DAT reading comprehension score increased it by 0.12 ± 0.05.

Significant predictors of a B+ or higher grade in the first year dental school physiology course also included undergraduate school rigor (*P* < 0.01), DAT total science score (P < 0.01), and DAT reading comprehension score (*P* < 0.05). Achieving a B+ or higher grade in the first year dental school biochemistry course was related to having taken an undergrad physiology course (*p* < 0.05) and DAT total science score (P < 0.01).

## Discussion

The OHS master’s program at Boston University has previously been shown to be an excellent credential enhancing pre-dental pipeline program [12]. This study demonstrated that a strong performance in the rigorous OHS master’s program, not a student’s UGPA, was a predictor of successful admission into dental school . Extending these results, we report that strong DAT science and reading comprehension scores and attending a mid or highly rigorous undergraduate institution are positive predictors for a strong performance in the OHS program. Furthermore, OHS students earning a B+ or better in the dental school Physiology and Biochemistry courses taken while enrolled in OHS as a master’s student were more successful in matriculating to dental school. Even with weaker admissions credentials (UGPA), OHS graduates who matriculated to BU’s School of Dental Medicine performed equally as well as traditional dental students during their first and second year [12] supporting similar results by Holmes et al. [14].

Many studies have sought to clarify admissions criteria that will predict success in medical, dental and doctoral programs. In graduate education, some schools have begun to move towards removal of the GREs from admission requirements [2]. In particular, doctoral programs in the biomedical sciences have begun to evaluate the need for the GRE in admissions and are moving towards a review based on research and academic performance. The study by Park et al. [9] demonstrates that GRE scores only weakly associate with academic success in such a doctoral program and this is further supported by others at University of North Carolina [15] and Vanderbilt [16]. Studies abroad reported of the usage of non-cognitive parameters in admissions. In the UK, changes to the UK Clinical Aptitude Test to include a situational judgement component have taken place to assess non-cognitive components of admission to reduce bias and assess non-cognitive traits [17, 18] whereas in dental school admissions, a study by Laia et al. cautioned against using test scores only [19]. Park et al. reported that school rigor or school competitiveness combined with research exposure were found to be strong predictors of success in a biomedical sciences doctoral program [9]. Similarly, our result suggest that attending a strong undergraduate institution correlates with success in the rigorous OHS master’s curriculum while students from less rigorous institutions may struggle. Since the major selected and the number of sciences courses did not correlate with success in OHS, we conclude that school rigor may be more important than UGPA in admissions decisions. With this being said, however the MCATs and DATs are still an important piece of the admissions [6, 7, 20].

Our results support the finding that credential-enhancing master’s programs such as OHS can serve to prepare students for success in dental school. We have shown this at BU’s School of Dental Medicine but also have tracked OHS graduates who have enrolled in dental schools across the country such as NYU, Tufts, Columbia and Meharry; students completing their dental education at these schools have had equal success. Other dental schools have reported success of students entering dental school following completion of master’s program such as that at College of Dental Medicine-Arizona at Midwestern University [21] and those at Tufts and Barry University Biomedical Sciences Master’s programs [22].

Since part of OHS’s mission is to increase diversity and serve as a pipeline program, one of our goals is to seek other predictors of success that do not compromise students from underrepresented groups and those from disadvantaged backgrounds from bias. Studies have shown that overreliance on GRE scores may compromise admission of students historically underrepresented in STEM [23]. A systematic review of medical school admissions from 2000 to 2014 looked at the importance of interviews including multiple mini-interviews as well as situational judgement tests in addition to test scores and found in general that aptitude tests are considered barriers for applicants from underrepresented groups [24]. Similar results have been shown among dental school applicants [25]. One study further looked at AADSAS application timing and found that students from underrepresented groups tend to apply later in the application cycles and thus are placed at a disadvantage just due to the delay, suggesting there are many approaches that can be implemented to improve the number of applicants and enrollees from underrepresented groups [26].

Keeping this in mind, it would be beneficial if dental schools could more accurately predict which applicants will be successful in the rigorous curriculum with tools other than the DATs that may be a barrier for applicants from underrepresented groups. Our results reported here support the finding that a combination of strong coursework at a rigorous undergraduate (or graduate) institution might be one such tool for the evaluation of cognitive abilities rather than DAT scores. Additionally, means of support must also be in place once students arrive on campus. Schools like Texas A&M Health Science Center Baylor College of Dentistry have worked to improve enrollment and retention of students from underrepresented groups through strong mentoring programs [27]. Elks et al. at Morehouse Medical School further supported the need to look beyond test scores and have programs in place to strengthen mentoring and academic support to improve academic performance and success on board exams [28] as well as Chaviano-Moran at Rutgers School of Dental Medicine [29]. Similarly, mentoring and professional development are a large part of OHS student success with our near-peer program [12]. Supporting students with academic aids, tutors, review sessions in addition to strong peer-peer mentoring and professional develop all contribute in preparing them for the rigors of dental school both academically and professionally.

Will the DATs and the MCATs still be required for admission to professional school? The answer is likely yes, at least in the near future. However, can programs like OHS and other post-baccalaureate credential enhancing master’s programs serve to provide a data-driven approach to admissions by using undergraduate courses taken and school rigor as an alternative approach to standardized test scores? We believe our results here support this conclusion. Previous studies have looked at undergraduate prerequisites and changes to some admission’s requirements have been made [30, 31]. Such trends may be identifiable at individual institutions and support transitioning to using such tools rather than test scores. Specifically, for the master’s program at BU, Physiology seems to fit the criteria although at this point the data is not generalizable more broadly. Physiology may be a predictor due to the conceptual nature of the course similar to the reading comprehension DAT score. Plouffe et al. found similar results with regard to Chemistry test scores in a Canadian dental school suggesting more conceptual courses such as Chemistry and Physiology may be able to serve as predictors [6]. Further, the results presented here suggest that students from less rigorous undergraduate institutions may just need more preparatory work such as a master’s program [12]. This is extremely relevant to students from underrepresented groups who may have had to work throughout their undergraduate training. Although many of these pipeline programs force applicants to accrue additional debt, it is necessary. Loan-forgiveness programs and scholarships can help to defray the costs. Furthermore, we feel that elucidating additional admission’s predictors to assist with pipeline programs is applicable to other postgraduate professional training programs. Understanding alternative predictors of success, we can work to develop guidelines to reduce some of the intrinsic bias in admissions, as it is imperative that committees continue to look beyond the DATs (or the MCATs) to other factors in order to decrease the tremendous gap between professionals from underrepresented groups and the current population they serve.

## Conclusion

We report here that strong test scores, attending a mid or highly rigorous undergraduate institution and completion of an undergraduate Physiology course are positive predictors of future success in the first year of dental school didactic coursework. Further, having students complete a post-baccalaureate master’s program is one way to further prepare students for the rigors of dental school. We hope these findings will further refine and guide admission’s decisions and improve recruitment to, and future success of, graduate student’s pursuit of professional school and in particular pipeline programs. Understanding alternative predictors of success may help to reduce the intrinsic bias among applicants from underrepresented groups and continue to look beyond the DATs (or the MCATs for pre-medical students) to decrease the tremendous gap between professionals from underrepresented groups and the population they serve.

## Data Availability

The datasets used and/or analyzed during the current study are available from the corresponding author on reasonable request.

## Abbreviations

BCP: Biology, Chemistry, Physics
BCPM: Biology, Chemistry, Physics, Math
BU: Boston University
DAT: Dental Admissions Test
GPA: Grade point average
GRE: Graduate record exam
MCAT: Medical College Admissions Test
MS: Masters of Science
OHS: Oral Health Sciences
UGPA: Undergraduate GPA
